# Relationships between College Students’ Sedentary Behavior, Sleep Quality, and Body Mass Index

**DOI:** 10.3390/ijerph18083946

**Published:** 2021-04-09

**Authors:** Wenxi Liu, Qin Yuan, Nan Zeng, Daniel J. McDonough, Kun Tao, Qingwen Peng, Zan Gao

**Affiliations:** 1 College of Kinesiology and Health Science, Huaihua University, Huaihua 418008, China; liux4443@umn.edu (W.L.); taokun0506@163.com (K.T.); pqw1273@163.com (Q.P.); 2 School of Kinesiology, University of Minnesota Twin Cities, Minneapolis, MN 55414, USA; mcdo0785@umn.edu; 3 Health Sciences Center, University of New Mexico, Albuquerque, NM 87106, USA; NZeng@salud.unm.edu

**Keywords:** accelerometer, sedentary behavior, sleep, body mass index

## Abstract

Purpose: Sedentary behavior (SB), sleep efficiency (SE), sleep duration (SD), and body mass index (BMI) are crucial determinants of an individual’s health. However, empirical evidence regarding associations between these factors in young adults living in China remains unknown. Therefore, the purpose of this study was to examine the relationships between accelerometer-measured SB, SE, SD, and BMI in Chinese college students. Methods: Two-hundred and twenty college students (115 females, *Mean_age_* = 20.29 years, SD = 2.37) were recruited from a south-central Chinese university. Participants’ SB (daily % time spent in SB), SE (number of minutes of sleep duration/number of minutes in bed), and SD were assessed via wrist-worn ActiGraph GT9X Link accelerometers for one week. Body weight was measured using a digital weight scale, height was measured using a stadiometer, and BMI was calculated as weight (kg)/height (m^2^). Results: Participants’ average time spent in SB was 76.52% (SD = 10.03), SE was 84.12% (SD = 4.79), and BMI was 20.67 kg/m^2^ (SD = 3.12), respectively. Regression analyses indicated that SB (β = −0.17, *p* = 0.01) and BMI (β = −0.20, *p* < 0.01) negatively predicted SE. In addition, BMI negatively predicted SD (β = −0.22, *p* < 0.01). Conclusion: Prolonged SB (e.g., screen viewing, smartphone use, and computer playing) and higher BMI may link to shorter sleep duration and lower sleep efficiency in Chinese young adults. Future randomized controlled trials are needed to further confirm these findings. Given that increased BMI status and SB may relate to adverse health outcomes, more population-based intervention strategies seeking to lower BMI and reduce SB (e.g., nutrition education and physical activity promotion) are needed in this population.

## 1. Introduction

The prevalence of sedentary behavior (SB) has reached epidemic levels, and is now a major public health problem [1,2,3,4]. SB is defined as any waking behavior characterized by an energy expenditure of less than 1.5 metabolic equivalents (METs), such as sitting, reclining, or lying [5]. Regardless of one’s physical activity (PA) levels, SB has been observed to be an independent risk factor for the development of chronic morbidities, such as overweightness/obesity, cardiovascular diseases, type 2 diabetes, and premature mortality [1,6,7,8,9,10]. Indeed, quantitative reviews have found that SB accounted for 3.8% of all-cause mortality, and also observed strong associations between SB and obesity, cardiovascular disease, and type 2 diabetes [11,12].

Sleep quality is important for health. Poor sleep quality has been associated with a number of negative health consequences, such as obesity, depression, cardiovascular disease, and all-cause mortality [13]. The guidelines recommend that adults should obtain at least 7–9 h of sleep to maintain overall health and well-being [14]. However, the prevalence of poor sleep quality remains high, as 40% of young adults have reported having problems with sleep, such as lower sleep efficiency (i.e., the ratio of time sleeping divided by total time in bed), decreased sleep duration (i.e., shorter overall sleeping time), longer sleep latency (i.e., the amount of time it takes to fall asleep), and increased sleep disturbances (i.e., number of instances in which one wakes up) [15]. 

Previous studies have indicated that prolonged SB has been associated with poor sleep quality [14,15,16,17,18,19,20,21]. Moreover, a recent review found that prolonged SB is associated with an increased risk of insomnia and sleep disturbances [1]. In addition, a recent randomized controlled trial examined the effects of SB on sleep quality by randomly assigning active young adults into a SB intervention (increasing sedentary behavior intentionally) or a control group for one week, and found that young adults who increased their time in SB experienced significant decreases in sleep quality [16]. In addition, compiling research has indicated significant associations between SB, sleep quality, and obesity [22,23,24,25]. One recent study evaluated college students’ sleep quality and body fat mass, and found that poor sleep efficiency was strongly associated with increased fat mass in both men and women [23]. Another study examining young adults’ SB and sleep quality found that young adults who watched more than three hours of TV/screen viewing were at greater risk for sleep problems in early adulthood than those who watched less than three hours of TV/screen viewing per day [26]. The investigators concluded that prolonged SB was closely linked to poor sleep quality and increased risk of obesity.

Previous studies have indicated significant increases in SB and reductions in PA during the transition from high school to college [27]. Due to the nature of the higher education system, students may spend more time in SB, such as sitting in the classroom, reading in the library, and studying at home [28]. In China, many studies have been conducted in order to promote PA among this population; however, SB as an independent risk factor of health promotion has been rarely studied. Recently, a systematic scoping review synthesized 162 studies regarding the prevalence of SB in the Chinese population, and revealed a high prevalence of SB across various population groups [2]. However, only 3.7% of studies measuring SB adopted device-based instruments (i.e., accelerometer), and most of the studies (64.8%) were based on self-report questionnaires. In addition, the assessment of sleep quality among young adults primarily relied on self-reported surveys [18]. The lack of device-based measurements for SB and sleep in the Chinese college population limits the strength and generalizability of findings from the preceding study. In order to measure more precisely, more studies using device-based measurement on SB and sleep are warranted.

To fill the research gap regarding the examination of the relationships among SB, sleep quality, and weight status in young adults in China, the purpose of this study was to examine the relationship among accelerometer-measured SB, sleep quality, and weight status. We hypothesized that SB and weight status would negatively associate with sleep quality (i.e., lower sleep efficiency and shorter sleep duration) among Chinese college students. Compared to previous studies assessing SB and sleep quality using self-report questionnaires, the present study aimed to provide assessments with higher reliability by use of accelerometers and examine the relationship among SB, sleep quality, and weight status. The findings of this study will help health professionals and researchers in developing effective intervention programs for improving sleep quality and overall health among college students.

## 2. Materials and Methods

### 2.1. Participants

A total of 220 college students (115 females; *Mean_age_* = 20.29 years, SD = 2.37; *Mean*_BMI_ = 20.67 kg/m^2^, SD = 3.12) from one university in south-central China were recruited for this study, and data were collected in June 2017. Inclusion criteria were (1) 18–25 years of age; (2) registered student at the study university at the time of data collection; (3) no self-report or diagnosed physical or mental disability; and (4) willing to provide informed consent to participate in the study. 

### 2.2. Measurements

For sedentary behavior (SB), an ActiGraph GT9X Link (ActiGraph; Pensacola, FL, USA) accelerometer was used to capture participants’ time in SB. This accelerometer has been used in many studies, and has demonstrated good reliability and validity in assessing adults’ SB [29,30]. Accelerometers were initialized based on a 30 Hz sampling rate, and SB was defined as 0–99 counts per minute [31]. The analyses for SB were based on a 60 s epoch length. To validate wear time, the non-wear time was defined as intervals of at least 60 consecutive minutes of zero activity counts, allowing for up to two consecutive minutes of counts between 1 and 100 counts [32]. Participants were asked to wear the accelerometer on their non-dominant wrist for seven consecutive days (including at least two weekdays and one weekend day), as suggested from previous field-based accelerometer research [33]. Data were imported to and processed in ActiLife software (Version 6.13.3; Pensacola, FL, USA). Participants were asked to wear the accelerometers for seven consecutive days without removing the device [34]. Participants’ daily average percentage of SB was calculated as the study outcome.

For sleep efficiency (SE) and sleep duration (SD), the same accelerometers were also used to assess participants’ SE and SD. The Sadeh algorithm was used to determine waking and sleep movements, which has demonstrated good validity and reliability [35]. SD was defined as the total true sleep time. Participants’ SE was calculated as total sleep duration divided by total time in bed. Poor sleep was defined as a night of total sleep time less than seven hours or SE below 85% [19]. Sleep diaries (records of time in bed and out of bed) were used to facilitate the sleep analysis. 

For body mass index (BMI), height was measured using Seca 213 stadiometer (Seca; Hamburg, Germany), and body weight was measured using Seca digital weight scale (Seca; Hamburg, Germany). When measuring height, participants were asked to remove their shoes and stand on the scale with their head oriented in the Frankfurt plane. Moreover, participants’ weight was measured in the morning and under standard conditions. Participants’ body mass index (BMI) was calculated as weight (kg) divided by the square of their height (m^2^) [36]. BMI has been widely used to determine individuals’ weight status and has been categorized as normal weight (BMI = 18.5–24.9 kg/m^2^), overweight BMI 25.0–29.9 kg/m^2^), or obese (BMI ≥ 30 kg/m^2^) [37].

### 2.3. Procedure

The present study was approved by the study university’s ethics committee (20170606P1), and written informed consent was obtained from participants prior to any data collection. The researchers collected all anthropometric data during regularly scheduled college physical education classes. Participation was voluntary, and no extra credit was awarded to participating students. Accelerometers were required to be worn at all times and during all activities, except for swimming or taking a shower. The accelerometer-measured SB and sleep data (sleep efficiency and sleep duration) were collected during the seven-day wear period. 

### 2.4. Data Analysis

Prior to data analyses, the validity of the SB data collected from participants was examined. In detail, participants with at least two weekdays and one weekend day of SB and sleep data were considered as valid for analysis. After the initial examination, all participants met the validation criteria. First, descriptive statistics were used to describe the participants’ demographic outcomes (e.g., age, gender), BMI, average daily SB (%), SE (%), and SD (minutes). Second, Pearson product–moment correlations were computed to examine the relationships between SB, SE, SD, and BMI. Third, multiple linear regression was used to examine the predictive associations among the study outcomes. Statistical significance was set at 0.05. Data were analyzed by Statistical Package of the Social Sciences (SPSS Version 27; IBM Inc., Armonk, NY, USA).

## 3. Results

Table 1 shows the participants’ characteristics and study outcomes. Descriptive statistics indicated that participants’ average time spent in SB was 76.52% (SD = 10.03), representing approximately 9.18 h spent in a sedentary position. SE was 84.12% (SD = 4.79), and SD was 341.67 min (SD = 80.65), indicating an average of 5.69 h (SD = 1.34) per day of sleep. Participants’ average BMI was 20.67 kg/m^2^ (SD = 3.12), indicating an overall healthy weight status of the present sample. Notably, we also compared the SB, SE, SD, and BMI between males and females. The results indicated that there was only one significant difference between gender in relation to BMI (*p* = 0.005, Cohen’s *d* = 3.07). Males (*Mean* = 21.29 kg/m^2^, SD = 3.54) had higher BMIs compared to females (*Mean* = 20.11 kg/m^2^, SD = 2.56) in this study sample.

Table 2 shows the correlations between study outcomes. Overall, significant relationships were observed among SB and SE (*r* = −0.16), SD and SE (*r* = 0.31), BMI and SE (*r* = −0.19), and BMI and SD (*r* = −0.22). In detail, SB was negatively correlated with SE (*p* = 0.02), SD was positively correlated with SE (*p* < 0.01) and BMI was negatively correlated with SE (*p* = 0.01) and SD (*p* < 0.01). Notably, the strength of the correlations among variables was weak. There were no significant correlations among the other outcomes. After controlling for gender, the significant correlations among variables were reexamined. Specifically, SB was negatively correlated with SE (*r* = −0.15), and BMI was negatively correlated with SE (*r* = −0.19) and SD (*r* = −0.23). Table 3 shows the results of the multiple linear regression analyses. In detail, SB, SD, and BMI were observed as predictors for sleep quality. Specifically, SB (*β* = −0.193, *p* = 0.01), SD (*β* = −0.299, *p* < 0.01), and BMI (*β* = −0.136, *p* < 0.01) negatively predicted SE and accounted for 15% of the observed variance. In addition, BMI negatively predicted SD (β = −0.222, *p* < 0.01), which explained 5% of variance. 

## 4. Discussion

The present study used accelerometers to assess SB and sleep quality and examined the relationships between Chinese college students’ time spent in SB, SE, SD, and BMI. We observed that college students spent about 77% of their daily wake time in SB, which account for 9.18 h of SB per day. Similar findings were also reported in a recent systematic review study [2]. The results indicated that young adults reported more than 6 h (range: 6–10 h) of SB per day in China [2]. In addition, an international study that included 10 countries examined accelerometer-measured SB among adults, and found that the average time spent in SB was about nine hours [38], which is consistent with the time in SB observed in the current study. Furthermore, one study examining SB and SB-related correlates among Chinese college students observed a daily screen time prevalence of 73% of wake time [39]. In addition, the study indicated that greater amounts of screen time were associated with sitting time, and the high use of Internet among college students was associated with higher BMI [39]. Similarly, another study reported that the excessive use of smartphones significantly contributed to time spent in SB among Chinese college students. Indeed, young adults enter college with more autonomy than during high school, and as a result, most college students spend their leisure time in SBs, such as screen viewing, smartphone use, and playing on computers. The alarming prevalence of SB among college students warrants health professionals’ and researchers’ attention, as evidenced by the fact that prolonged SB is an independent risk factor associated with adverse health consequences, such as overweightness/obesity, poor sleep quality, cardiovascular diseases, type 2 diabetes, etc. [40,41,42].

Regarding sleep quality, the current study observed that participants had an overall SE of 84% which is below the recommended SE ratio of 85% [43]. In addition, we found that participants had an average of less than six hours of total sleep duration, which is also well below the recommended levels of at least seven hours of sleep per night for healthy adults [44]. Literature examining Chinese college students’ sleep quality correlates is sparse. Only one study has assessed college students’ sleep quality by using self-reported surveys, and it found that less than 26% of students reported having sleep problems, such as trouble falling to sleep, shorter overall sleep duration, and reoccurring sleep disturbances [45]. One meta-analysis reported an average of seven hours of sleep per night among Chinese college students; however, the included studies were all based on the self-reported sleep and wake time [46]. This study also found that the shorter sleep duration was associated with unhealthy sleep habits, such as smartphone use in bed and reading or watching media on electronic devices. A recent meta-analysis involving 18,619 medical students across 13 countries examined sleep-related problems, and found that the students averaged only six hours of sleep per night. Thus, the prevalence of poor sleep quality among college students warrants further investigation, and there is an urgent need to develop effective strategies by which to improve overall sleep quality in this population.

We hypothesized that SB may be negatively associated with sleep quality. In line with our hypothesis, the findings of the present study indicate that participants’ time spent in SB was negatively associated with their SE, indicating that the prolonged SB may lead to lower SE. This is in line with a previous study examining the associations among college students’ PA, SB, and sleep quality, which observed total sleep time to be negatively associated with SB, but there was no association between PA and sleep quality [19]. Their findings further suggest that poor sleep quality was more related to participants’ SB than their PA levels. Moreover, one meta-analysis examined the relationship between SB and sleep problems, and found that prolonged SB may lead to increased risk of insomnia and sleep disturbances. Notably, there are very limited experimental studies to further conclude the causal effect of SB on sleep issues. According to a recent meta-analysis examining Chinese college students’ sleep pattens, the prevalence of shorter sleep may be explained by several factors, such as late-night activities and early morning school demands, as well as smartphone use and screen viewing in bed before going to sleep [46]. Another study examining 1106 Chinese college students’ PA, screen time, and sleep quality observed that lower screen time was associated with greater sleep quality [39]. It appears that larger amounts of SB may be attributed to smartphone use and screen viewing (e.g., watching TV, computer use, and the use of other electronic devices) in Chinese college students. One way to combat SB is to increase PA time. Clear research evidence has suggested the beneficial effects of PA in reducing SB and improving overall sleep quality. However, one study examining Chinese college students’ PA and correlates found that students lacked motivation to engage in sufficient PA [27]. To this end, the observed negative association between SB and SE needs further experimental studies to conclude this relationship. As SB and sleep are important for health and well-being, future health practitioners and researchers are strongly encouraged to develop effective strategies for reducing the SB and improving sleep quality among college students.

In line with our second hypothesis, we also found that BMI was negatively associated with SE and SD, which indicates that higher BMI may lead to decreased SE and SD. Similar findings were found in a previous study [23] examining the relationship between sleep quality and obesity in college students, which found that lower SE was associated with higher fat mass. Notably, this study found that accelerometer-measured SE was positively associated with participants’ fat mass. However, given our study findings indicating an inverse relationship between BMI and SE, it appears that body composition and SE may have a mutual relationship and influence one another. Indeed, empirical studies have indicated that individuals with higher BMI are usually less active and report greater sedentary time, which is negatively associated with sleep quality [47,48]. Future investigations of the bi-directional relationships between sleep quality and body composition among college populations are needed. As previously discussed, it is very common that Chinese college students demonstrate unhealthy sleep habits, such as using smartphones, or reading or watching other electronic devices while in bed. Moreover, one review examined 50 epidemiological studies across populations around the world and observed a significant association between shorter sleep duration (i.e., under six hours per night) and increased risk of obesity [49]. Another explanation for this association may be derived from the hormonology perspective. Previous studies examining the association between regulation of ghrelin (a hunger-promoting hormone) and leptin (a hormone contributing to the perception of satiety) with sleep found an inhibitory effect of sleep on ghrelin secretion [50]. Simply stated, individuals’ secretion of ghrelin may increase with sleep deprivation, and may therefore lead to more energy intake during the night, which may ultimately contribute to an energy surplus, and thus, the elevated risk of developing overweightness/obesity. When considering the social and cultural norms in China, it is very common to consume food late at night, as reported in a recent meta-analysis examining the Chinese population regarding their sleep patterns and habits [46]. As the prevalence of overweightness/obesity continues to grow, college students not only should reduce their time spent in SB, but also need to establish good sleep habits in order to reduce their risk of overweightness/obesity. As an emerging topic among this population in China, future research should adopt longitudinal and experimental studies to further conclude causality and to develop effective strategies for improving SE and SD among Chinese college students.

The major strength of this study was the use of accelerometers to assess SB and sleep quality, as well as the examination of the relationships among SB, SE, SD, and BMI in Chinese college students. As most studies on this topic have relied on self-reported data, our findings provide more accurate and valid assessments that could help future studies develop more effective strategies or programs for promoting overall health in college students. Nevertheless, there are several potential limitations of the present study that must be acknowledged. First, the representativeness of study participants needs to be considered, as all participants were from one university in south-central China. Indeed, there are substantial differences in living habits, economic level, seasonal changes, and cultural contexts between the southern and northern parts of China. Thus, future studies with large-scale and more representative samples are warranted. Second, due to the cross-sectional nature of our study, the causal relationships between SB, sleep quality, and body composition could not be determined. Therefore, future longitudinal and experimental trials are strongly encouraged to further confirm the causality among SB, sleep quality, and body composition in the Chinese college student population. Third, we observed weak associations between our observed outcomes, and thus, caution is warranted when interpreting the findings of this study. Future studies with larger and more representative study samples are needed to more thoroughly examine these relationships. Lastly, although the use of accelerometers to determine SB and sleep provided more accurate assessments compared to self-reported surveys, caution is needed when processing the accelerometry data, because different epoch lengths may influence the sensitivity of detection between SB and PA [51] (e.g., a larger epoch length may lead to overestimation of SB). Our study used a 60 s epoch length when processing the accelerometry data, which may have overestimated the time spent in SB in this study. Notably, the 60-s epoch length has been used widely in many studies for assessing SB and sleep in free-living conditions [52]. Future studies are encouraged to use lower epoch lengths, (e.g., 1 s) [53] to process the data more accurately.

## 5. Conclusions

In this cross-sectional study, we found that both SB and BMI were negatively associated with sleep quality. The prolonged SB (e.g., screen viewing, smartphone use, and computer playing) and higher BMI status may lead to shorter sleep duration and lower sleep efficiency. As increased BMI and SB are linked to adverse health outcomes, more population-based prevention strategies seeking to reduce SB and improve sleep quality (e.g., lifestyle modification and PA promotion interventions) are needed in this population. 

## Figures and Tables

**Table 1:** Characteristics of study participants and descriptive statistics of study outcomes (*n* = 220).

	Mean	SD
Age (year)	20.3	2.4
Height (cm)	164.8	10.0
Weight (kg)	57.8	13.3
BMI (kg/m^2^)	20.7	3.1
Sedentary Behavior (%)	76.5	10.0
Sleep Efficiency (%)	84.1	4.8
Sleep Duration (minute)	341.7	80.7

Note: BMI, body mass index; SD, standard deviation.

**Table 2:** Correlation analyses among study outcomes.

	1	2	3
1. Sedentary Behavior	-		
2. Sleep Efficiency	−0.157 *	-	
3. Sleep Duration	0.096	0.311 **	-
4. BMI	−0.128	−0.192 **	−0.222 **

Note: ** correlation is significant at the 0.01 level (2-tailed); * correlation is significant at the 0.05 level (two-tailed); BMI, body mass index.

**Table 3:** Regression analyses among study outcomes.

Dependent Variables	Independent Variables	*β*	*R* ^2^	*p*
Sleep Efficiency	BMI	−0.136	0.15	0.037
	Sedentary Behavior	−0.193		0.003
	Sleep Duration	0.299		<0.001
Sleep Duration	BMI	−0.222	0.05	0.001

Note: *β*, standardized regression coefficient; *R*^2^, proportion of variance that can be explained by independent variables; BMI, body mass index.

## Data Availability

The data presented in this study are available on request from the corresponding author. The data are not publicly available due to containing information that could compromise the privacy of research participants.

## Availability of Data and Material

The data was collected on paper and transferred to electronical media on a computer spreadsheet for export to statistical packages. The paper data was stored in a locked file cabinet within the principal investigator’s office for a minimum of three years after the last participant has completed data collection. Electronic media files of the data were also stored for a minimum of three years on the principal investigator’s computer and on a computer within the University of Minnesota’s Physical Activity Epidemiology Laboratory. Both computers were password-encrypted and were only capable of being accessed by the principal investigator. The data collected during the current study will not be released or shared with anyone aside from participants.
